# Supplementary material for: Structures of two LarA‐like nickel‐pincer nucleotide cofactor‐utilizing enzymes with a single catalytic histidine residue
Source: Protein Sci. 2025 Nov 12;34(12):e70362. doi: 10.1002/pro.70362 (PMC12611868; doi:10.1002/pro.70362)
Supplement: Supplementary file 1 — Data S1. Supporting Information. [file PRO-34-e70362-s001.docx]

Supplementary information

**Structures of two LarA-like nickel-pincer nucleotide cofactor-utilizing enzymes with a single catalytic histidine residue**

Santhosh Gatreddi,^1,2,*^ Sundharraman Subramanian,^2*^ Dexin Sui^2^, Tianqi Wang,^2^ Julian Urdiain-Arraiza,^3^ Benoît Desguin,^3^ Robert P. Hausinger,^1,2,‡^ Kristin N. Parent,^2,‡^ Jian Hu^2,4,‡^

^1^Department of Microbiology, Genetics, and Immunology, Michigan State University, MI, USA

^2^Department of Biochemistry and Molecular Biology, Michigan State University, MI, USA

^3^Louvain Institute of Biomolecular Science and Technology (LIBST), Université catholique de Louvain, B-1348 Louvain-La-Neuve, Belgium

^4^Department of Chemistry, Michigan State University, MI, USA

^*^Equally contributive to this work

^‡^Corresponding authors: Robert P. Hausinger ([hausinge@msu.edu](mailto:hausinge@msu.edu)), Kristin Parent ([kparent@msu.edu](mailto:kparent@msu.edu)), and Jian Hu ([hujian1@msu.edu](mailto:hujian1@msu.edu))

**
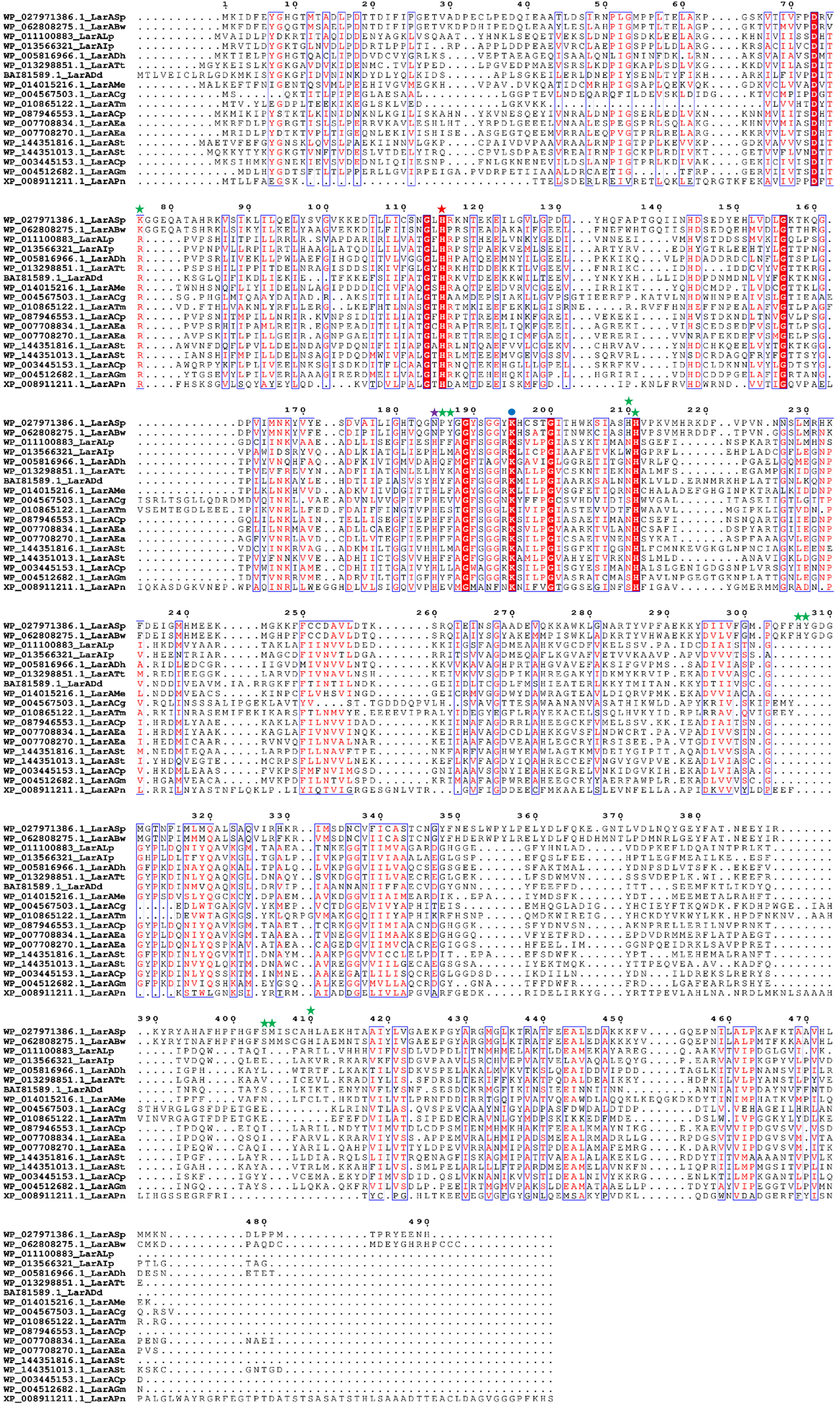
**

**Figure S1.** Multiple sequence alignment of LarA*_Bw_*, LarA*_Sp_*, and the LarAHs with known substrates. The catalytic histidyl group and the asparaginyl side chain substituting for the second histidine residue in LarA*_Bw_* and LarA*_Sp_* are highlighted with red and purple stars, respectively. Other active site residues are indicated with green stars. The lysine residue forming the thioamide bond with the NPN cofactor in LarAHs is labeled with a blue dot. The residue numbers are based on the amino acid sequence of LarA*_Sp_*. The protein IDs, species, and the primary activities are shown as follows. WP_027971386.1_LarA*Sp* from *Streptococcus plurextorum*, activity unknown; WP_062808275.1_LarA*_Bw_* from *Blautia wexlerae*, activity unknown; WP_011100883_LarA*_Lp_* from *Lactiplantibacillus plantarum*, lactate racemase; WP_013566321_LarA*_Ip_* from *Isosphaera pallida*, lactate racemase; WP_005816966.1_LarA*_Dh_* from *Desulfitobacterium hafniense*, malate racemase; WP_013298851.1_LarA*_Tt_* from *Thermoanaerobacterium thermosaccharolyticum*, malate racemase; BAI81589.1_LarA*_Dd_* from *Deferribacter desulfuricans*, hydroxyglutarate racemase; WP_014015216.1_LarA*_Me_* from *Megasphaera elsdenii,* hydrophobic 2-hydroxyacid racemase; WP_004567503.1_LarA*_Cg_* from *Corynebacterium* glutamicum, D-gluconate 2-epimerase, WP_010865122.1_LarA*_Tm_* from *Thermotoga maritima*, D-gluconate 2-epimerase; WP_087946553.1_LarA*_Cp_* from *Clostridium* pasteurianum, lactate racemase; WP_007708834.1_LarA*_Ea_* from *Enterocloster asparagiformis*, lactate racemase; WP_007708270.1_LarA*_Ea_* from *Enterocloster asparagiformis*, lactate racemase; WP_144351816.1_LarA*_St_* from *Sporomusa termitida*, malate racemase; WP_144351013.1_LarA*_St_* from *Sporomusa termitida*, hydrophobic 2-hydroxyacid racemase; WP_003445153.1_LarA*_Cp_* from *Clostridium pasteurianum*, hydrophobic 2-hydroxyacid racemase; WP_004512682.1_LarA*_Gm_* from *Geobacter metallireducens*, 2-hydroxyglutarate racemase; XP_008911211.1_LarA*_Pn_* from *Phytophthora nicotianae*, D-gluconate 2-epimerase.


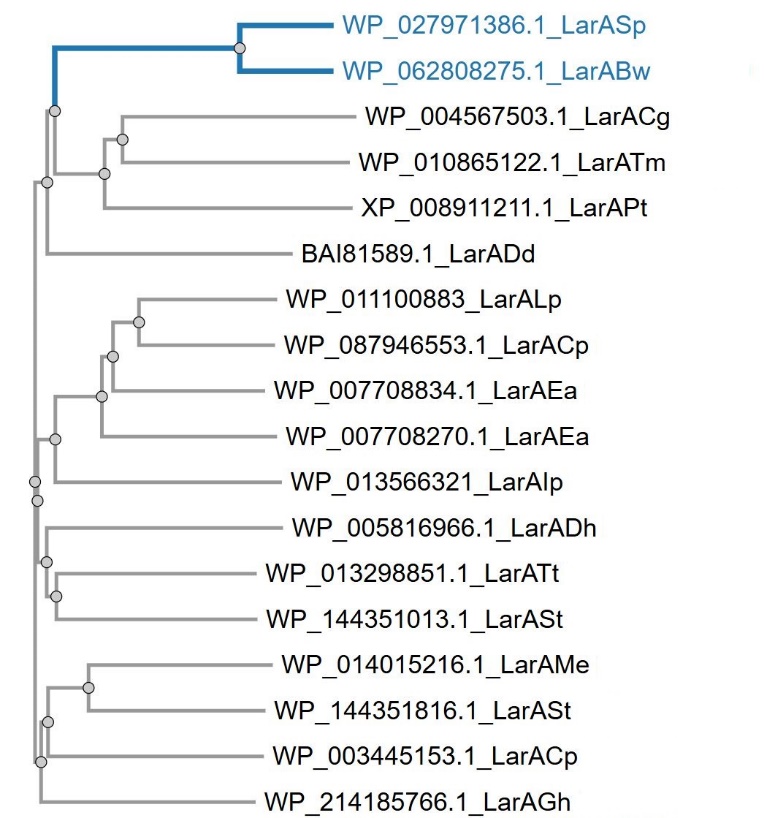


**Figure S2**. Phylogenetic analysis of LarAHs from the LarA family highlighting (in blue) the distinct branch for LarA*_Sp_* and LarA*_Bw_*. The latter proteins contain an asparagine residue at the active site, whereas other LarAHs contain a catalytic histidine residue at the same position.


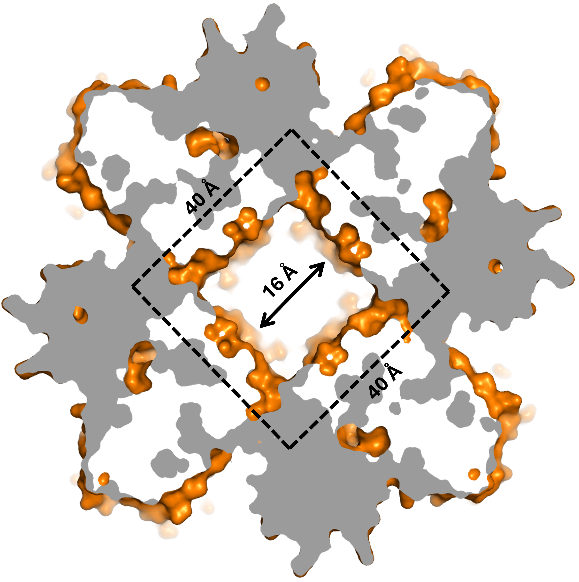


**Figure S3.** Cross-section view of the LarA*_Bw_* octamer in surface mode. The dashed frame indicates a square-shaped cavity with sides measuring ~40 Å within the octamer. The entrance size of the cavity is ~16 Å.


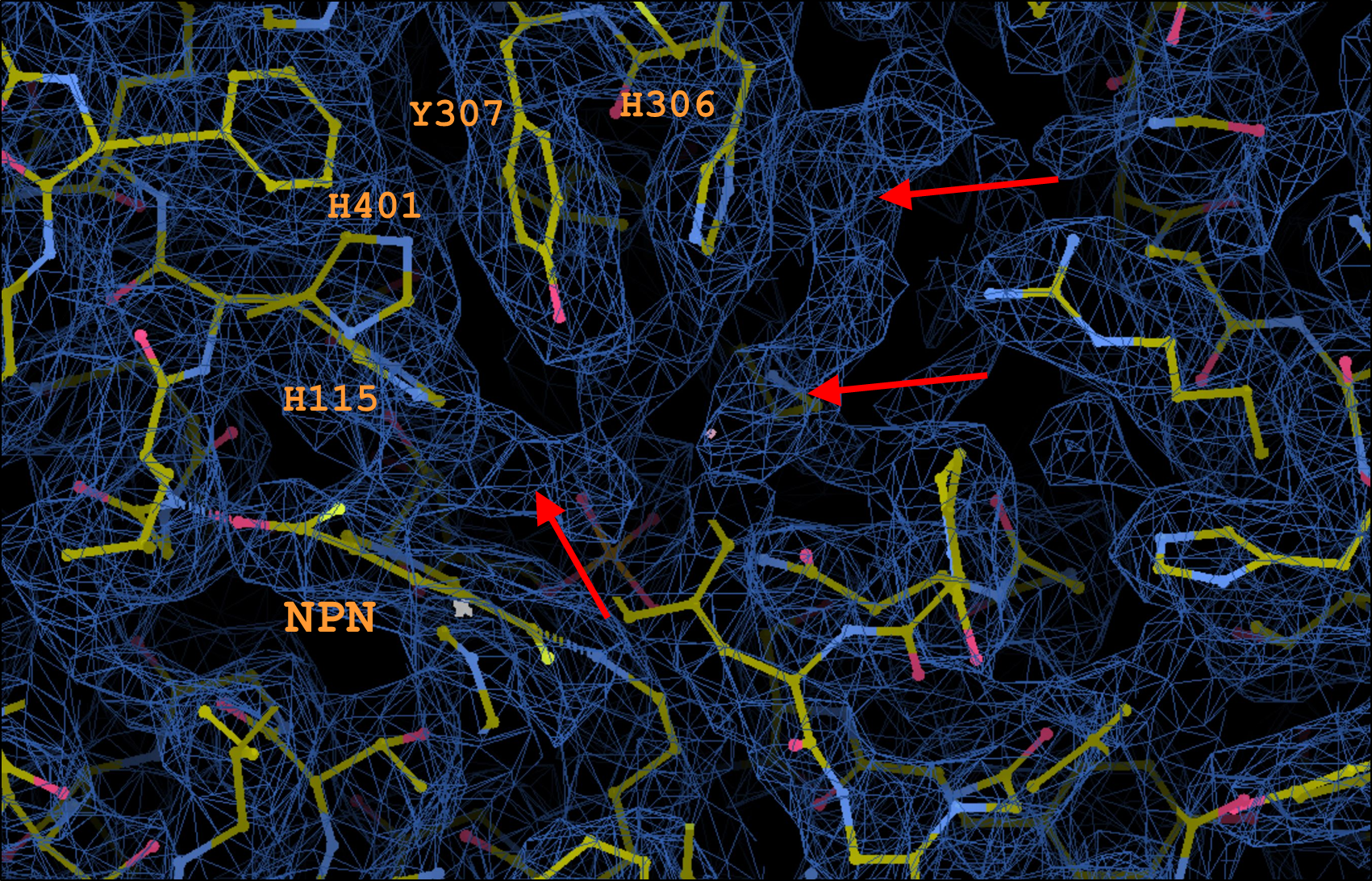


**FigureS4.** An unidentified ligand bound at the active site of LarA*_Sp_*_._ The electron densities of the ligand indicated by the arrows are close to several conserved active site residues. The contour level in Coot was set at 2.3 RMSD.


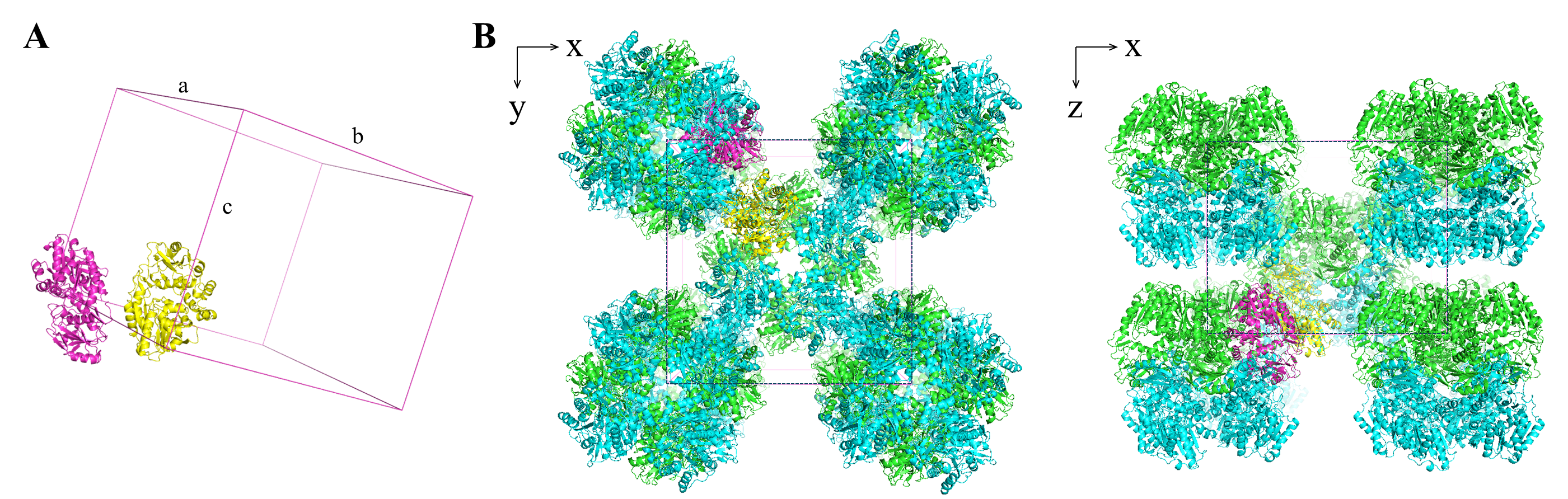


**Figure S5.** Crystal structure of LarA*_Sp_*. (**A**) Two polypeptide chains in one asymmetric unit of a unit cell. (**B**) Octameric assembly of LarA*_Sp_* in the crystal lattice with a I4 space group. The rectangles indicate the unit cell boundaries.

**
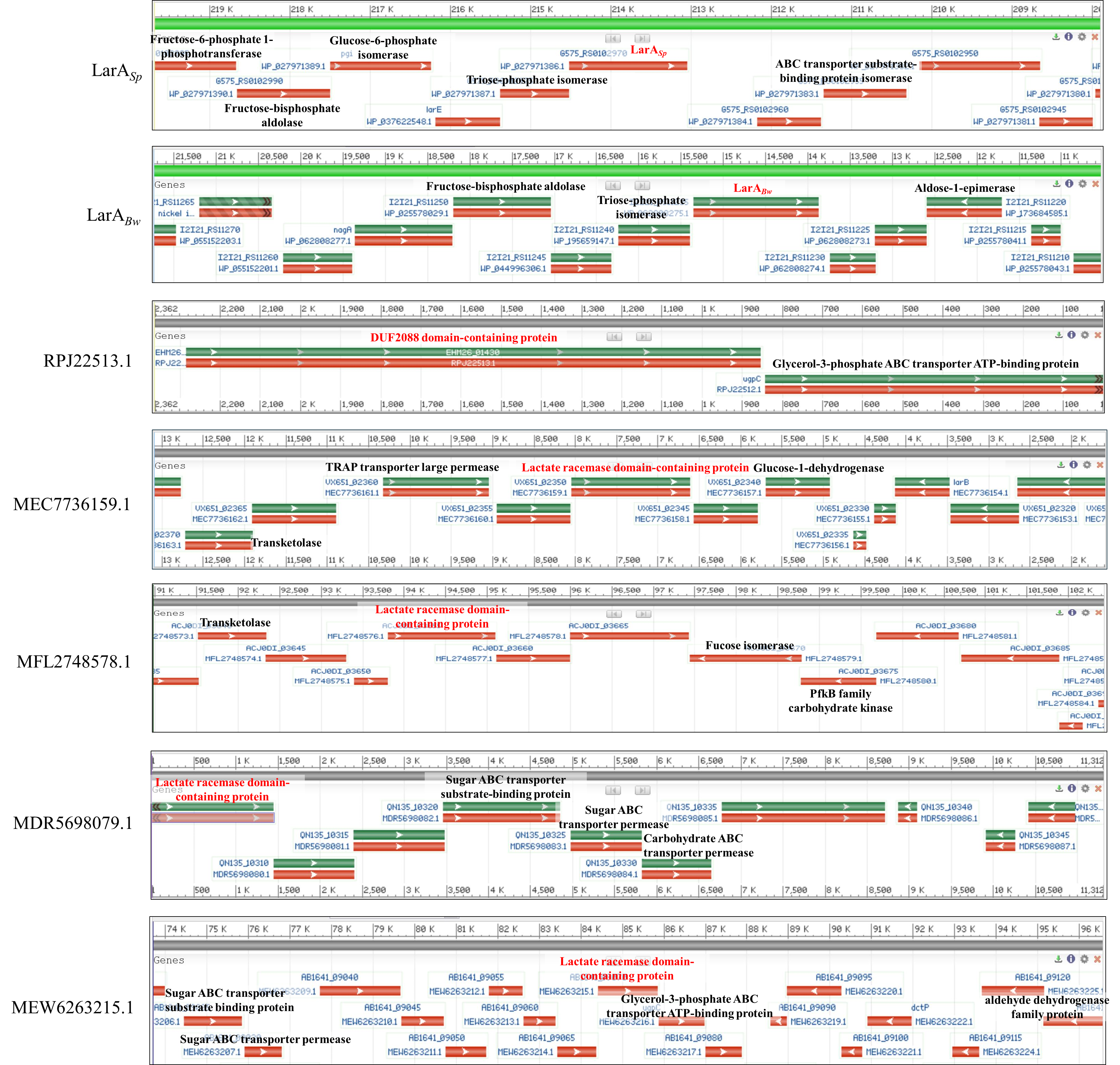
**

**Figure S6.** Genomic contexts of LarA*_Sp,_* LarA*_Bw_* and other LarAHs from the same subfamily. These LarAHs (from **Figure 1**) share as low as 37% sequence identity. The figures were retrieved from NCBI. Only the LarAH genes (in red) and the neighboring genes annotated to be involved in carbohydrate metabolism or transport are labeled.


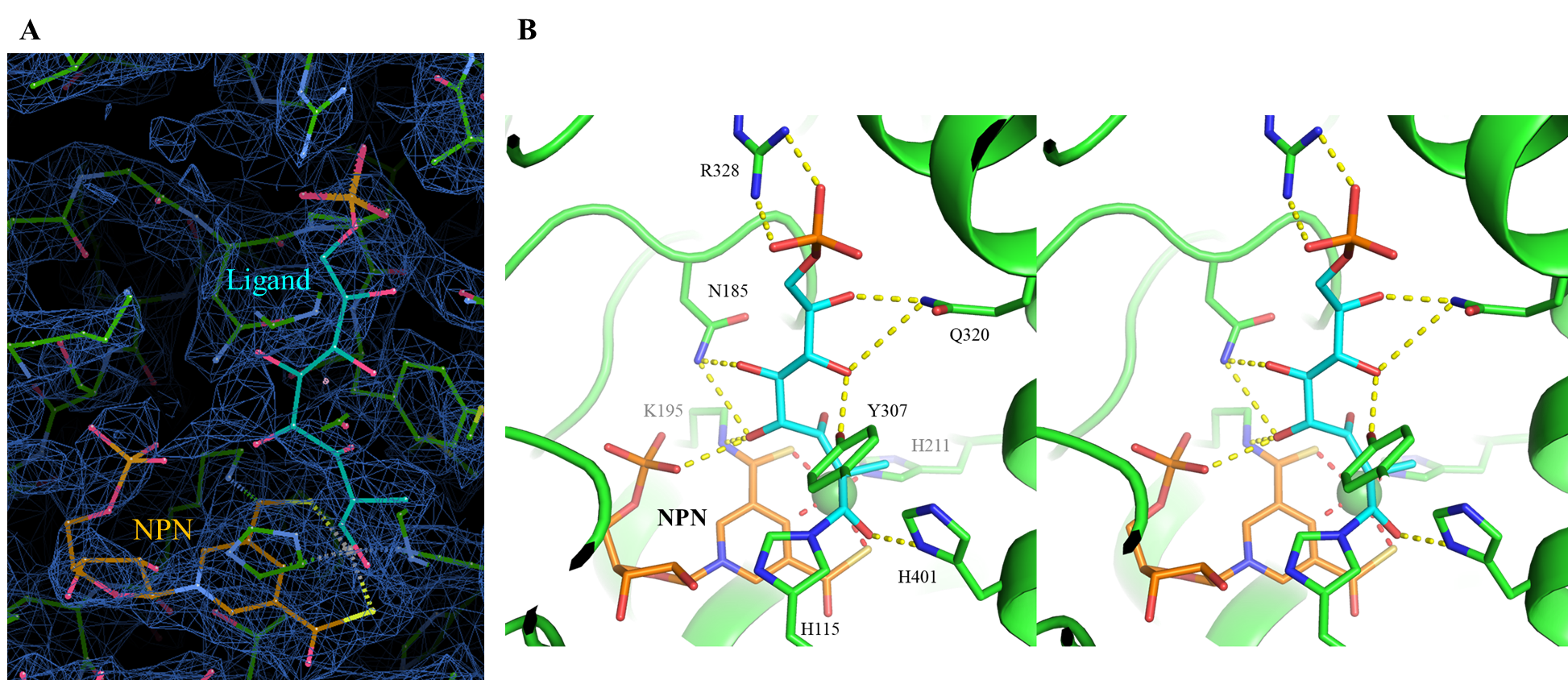


**Figure S7.** Modeling of the unidentified ligand in the active site of LarA*_Sp_*. (**A**) Modeling of a phosphorylated aldose with a formula of C_9_H_19_O_10_P. (**B**) Stereo view of the active site with the modeled ligand. The residues involved in hydrogen bond formation (yellow dashed lines) with the NPN cofactor or the ligand are labeled and shown in stick mode. The nickel ion is depicted as a green sphere. Note that this speculative modeling was used to estimate the size and shape of this ligand.


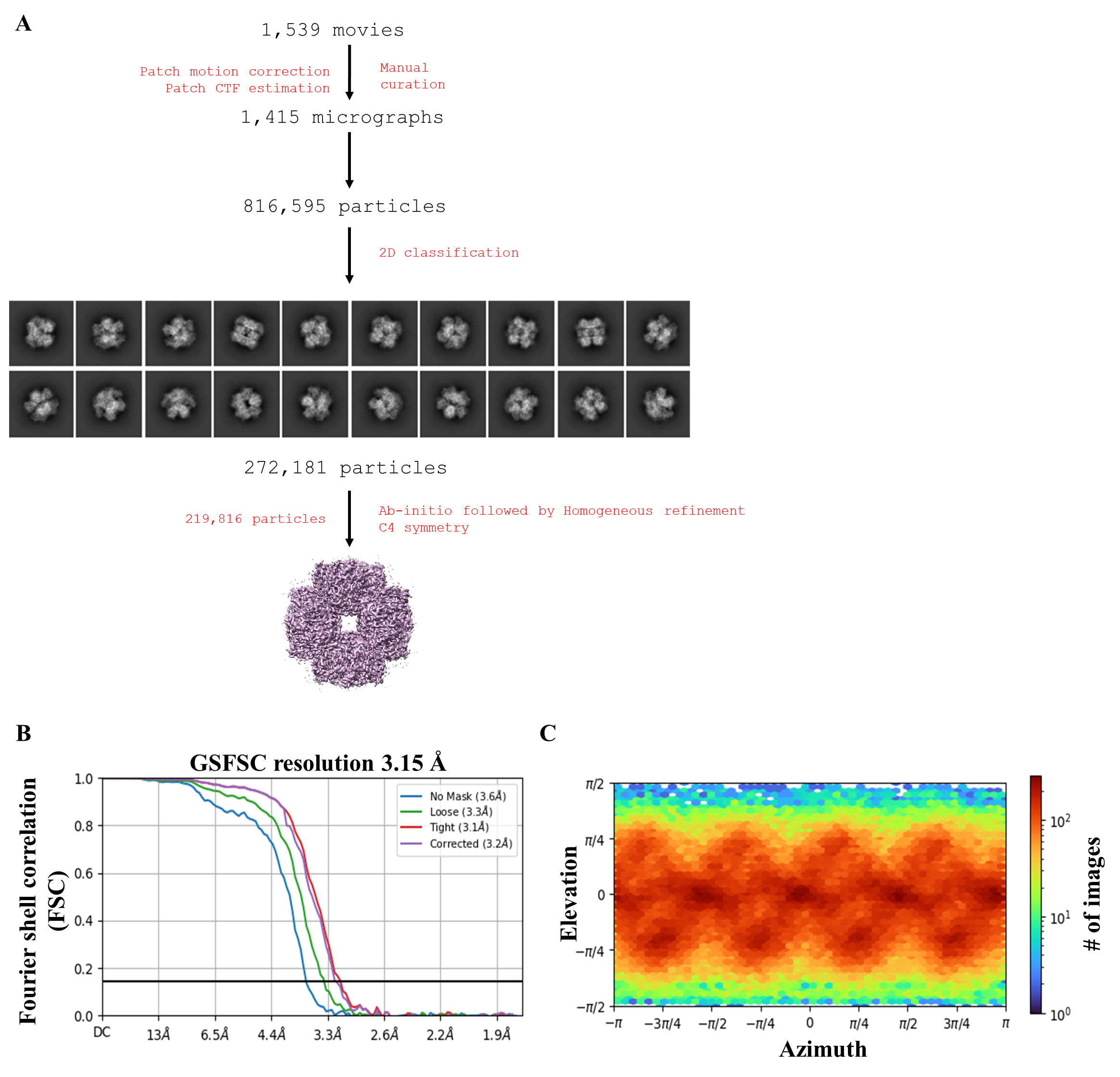


**Figure S8**. Cryo-EM data processing for LarA*_Bw_*. (**A**) Data processing workflow. (**B**) Gold standard FSC curve. (**C**) Orientation distribution plot.


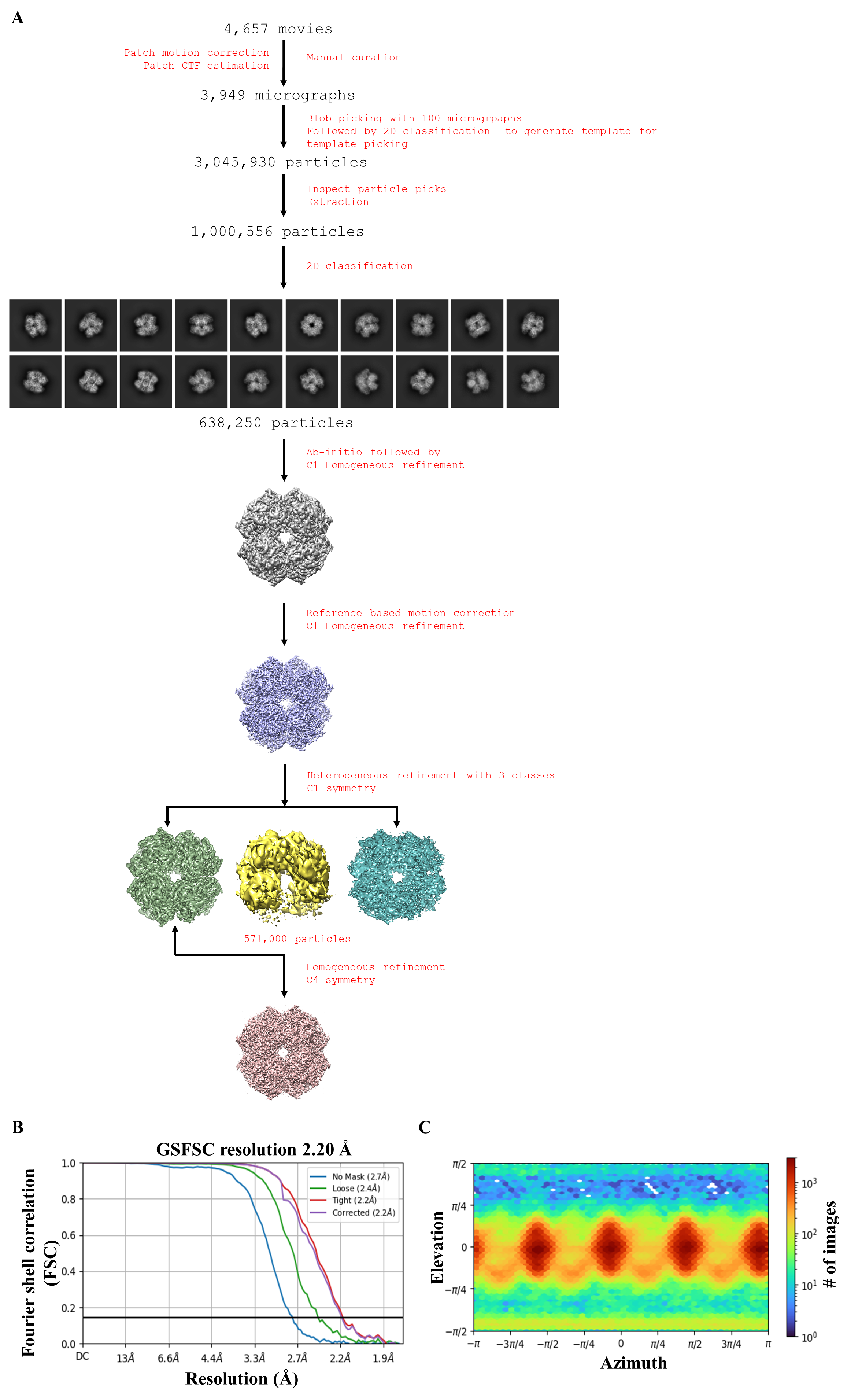


**Figure S9**. Cryo-EM data processing for LarA*_Sp_*. (**A**) Data processing workflow. (**B**) Gold standard FSC curve. (**C**) Orientation distribution plot.


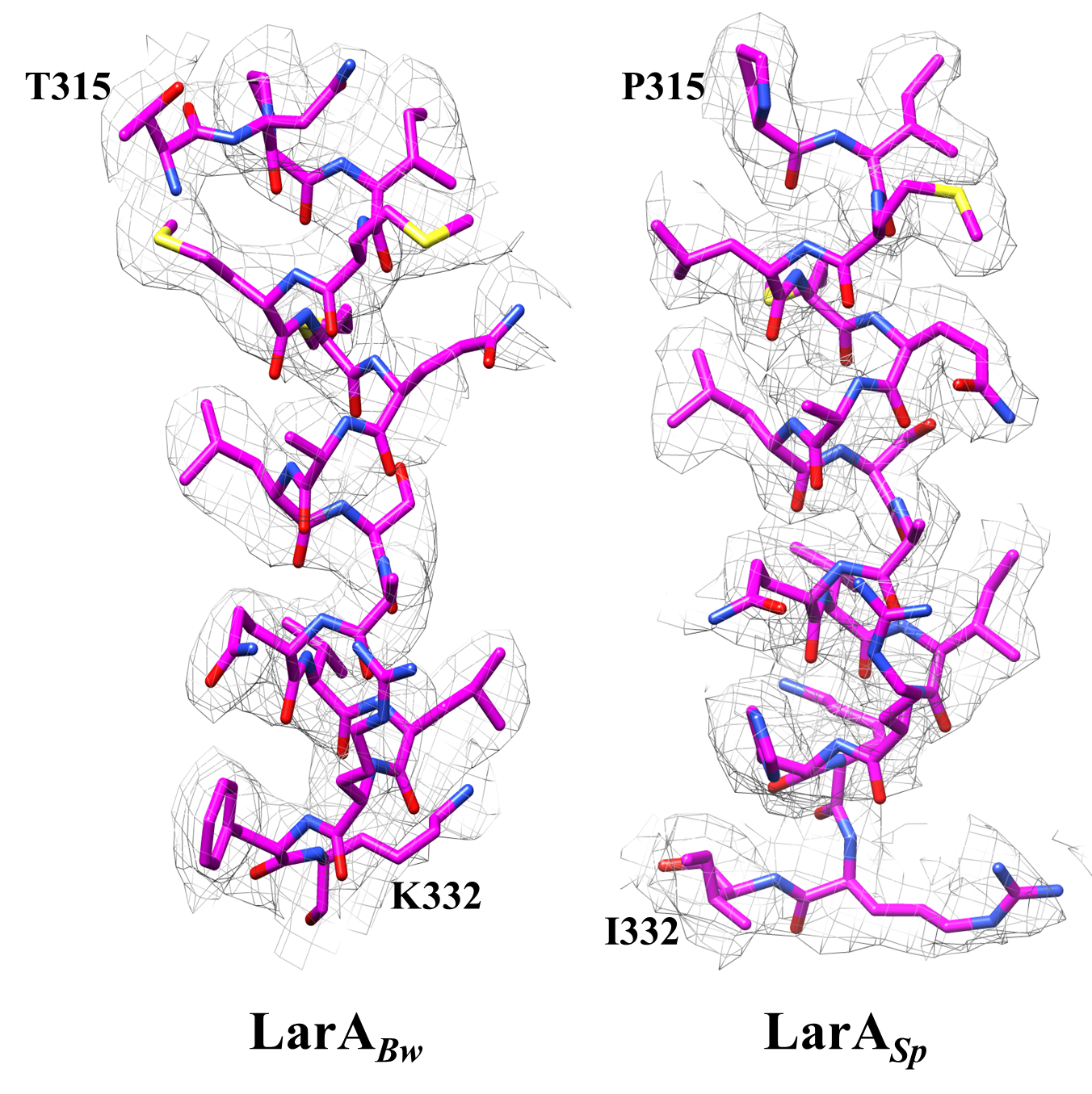


**Figure S10.** Representative cryo-EM density maps.

**Table S1.** Plasmids and primers used in this study.

| Strains, plasmids, or primers | Characteristic(s) or sequence |  | Source or reference |
| --- | --- | --- | --- |
| Strains |  |  |  |
| *L. lactis* NZ3900 | MG1363 derivative |  | ^1^ |
| *E. coli* BL21-Gold(DE3) |  |  | Invitrogen |
| Plasmids |  | |  |
| pGIR213 | Cm^r^; pGIR210 with DNA encoding LarA*_Sp_* | | This study |
| pET23b_LarA*_Bw_* | Amp^r^; pET23b with DNA encoding LarA*_Bw_* | | ^2^ |
| pGIR112_PciI | Cm^r^; pGIR112 with an introduced PciI site | | This study |
| pGIR112_LarA*_Bw_* | Cm^r^; pGIR112_PciI with DNA encoding LarA*_Bw_* | | This study |
| Primers |  | |  |
| LarAH37_A | aaaacatgtctaaaattgattttgaatacggtcatggg | | This study |
| LarAH37_B2 | aaatctagaatgattctcctcataacgaggcgtcatag |  | This study |
| PciI_F | ATAAATTATAAGGAGGCACTCAACATGTCCGTTGCAATTGATTTACCATATG |  | This study |
| PciI_R | CATATGGTAAATCAATTGCAACGGACATGTTGAGTGCCTCCTTATAATTTAT |  | This study |
| LarAH13_F | CATGCACATGTCCAGCAAATTTGATTTTGAGTATGGAC |  | This study |
| LarAH13_R | gatc GCT AGC acagcaacatggatgacgatgtcc |  | This study |
|  |  |  |  |

**Table S2.** Cryo-EM data collection, refinement and validation statistics.

| **Map**  EMD Identifier  PDB Identifier | **LarA*_Bw_***  EMD-72199  9Q3J | **LarA*_Sp_***  EMD-72200  9Q3K |
| --- | --- | --- |
| **Data collection and processing** | | |
| Magnification | 120,000 | 130,000 |
| Voltage (kV) | 200 | 200 |
| Electron exposure (e^–^/Å^2^) | 32.27 | 44.71 |
| Defocus range (µm) | -0.8 to - 3.0 | -0.8 to - 3.0 |
| Pixel size (Å) | 0.872 | 0.886 |
| Symmetry imposed | C4 | C4 |
| Number of micrographs | 1,539 | 4,657 |
| Number of particles | 219,896 | 571,110 |
| Map resolution (Å) | 3.15 | 2.20 |
| FSC threshold | 0.143 | 0.143 |
| **Refinement** | | |
| *Model composition* |  |  |
| Protein atoms | 28,832 | 30,332 |
| H_2_O molecules | 0 | 0 |
| Ni atoms | 0 | 8 |
| 4EY: P2TMN | 0 | 8 |
| *B-factors (Å^2^)* |  |  |
| Overall | 34.85 | 49.02 |
| Protein atoms | 34.85 | 48.99 |
| H_2_O molecules | 0 | 0 |
| Ni atoms | 0 | 103.5 |
| 4EY: P2TMN | 0 | 52.40 |
| *R.m.s. deviations* |  |  |
| Bond lenghts (Å) | 0.003 | 0.003 |
| Bond angles (°) | 0.551 | 0.566 |
| *Validation* |  |  |
| MolProbity score | 1.55 | 1.82 |
| Clashscore | 6.64 | 17.39 |
| Poor rotamers (%) | 0.23 | 1.12 |
| *Ramachandran* |  |  |
| Favored (%) | 96.88 | 97.88 |
| Allowed (%) | 3.12 | 2.12 |
| Outliers (%) | 0.00 | 0.00 |
| CC (volume) | 0.85 | 0.82 |

**Table S3.** Crystallographic statistics for LarA*_Sp_*.

| **Data collection** |  |
| --- | --- |
| Beamline | NSLSII 17-ID-2 FMX |
| Wavelength (Å) | 0.97934 |
| Space group | I4 |
| Unit cell a, b, c (Å);  α, β, γ (°) | 145.224, 145.224, 116.994  90.00, 90.00, 90.00 |
| ^a^Resolution (Å) | 34.23 – 3.108  (3.16-3.108) |
| ^a^Redundancy | 14.1 (14.8) |
| ^a^Completeness (%) | 100 (100) |
| ^a^*I/σI* | 7.3 (0.7) |
| ^a,b^*R_merge_* | 0.326 (4.635) |
| ^a,c^*R_pim_* | 0.090 (1.244) |
| ^d^CC_1/2_ | 0.995 (0.364) |
| **Refinement** |  |
| Unique reflections | 21,937 |
| Number of atoms | 7183 |
| Protein atoms | 7162 |
| H_2_O molecules | 21 |
| ^e^*R_work_/R_free_* | 0.222/0.282 |
| *B*-factors (Å^2^) | 94.6 |
| Protein atoms | 94.7 |
| H_2_O molecules | 72.9 |
| R.m.s. deviation in bond lengths (Å) | 0.0124 |
| R.m.s. deviation in bond angles (°) | 1.47 |
| Ramachandran plot (%) favored | 90.10 |
| Ramachandran plot (%) allowed | 9.80 |
| Ramachandran plot (%) outliers | 0.10 |
| Rotamer (%) outliers | 0 |
| PDB ID | 9Q2U |

^a^Highest resolution shell is shown in parentheses.

^b^*R_merge_* = ∑*_hkl_* ∑*_j_* |*I_j_*(*hkl*)-<I(*hkl*)>| / ∑_hkl_ ∑*_j_* *I_j_*(*hkl*), where *I* is the intensity of reflection.

^c^*R_pim_*=∑*_hkl_* [1/(N-1)]^1/2^∑*_j_* |*I_j_*(*hkl*)-<I(*hkl*)>| / ∑*_hkl_* ∑*_j_* *I_j_*(*hkl*), where N is the redundancy of the dataset.

^d^CC_1/2_ is the correlation coefficient of the half datasets.

^e^*R_work_* = ∑*_hkl_* | |*F_obs_*| – |*F_calc_*| | / ∑*_hkl_* |*F_obs_*|, where *F_obs_* and *F_calc_* is the observed and the calculated structure factor, respectively. R_free_ is the cross-validation R factor for the test set of reflections (5% of the total) omitted in model refinement.

**References**

1. de Ruyter, P.G., Kuipers, O.P., and de Vos, W.M. (1996). Controlled gene expression systems for *Lactococcus lactis* with the food-grade inducer nisin. *Appl Environ Microbiol* *62*, 3662-3667. 10.1128/aem.62.10.3662-3667.1996.

2. Chatterjee, S., Stupp, G.S., Park, S.K.R., Ducom, J.-C., Yates, J.R., Su, A.I., and Wolan, D.W. (2016). A comprehensive and scalable database search system for metaproteomics. *BMC Genomics* *17*, 642. 10.1186/s12864-016-2855-3.
